# Supplementary material for: β-catenin condensation facilitates clustering of the cadherin/catenin complex and formation of nascent cell-cell junctions
Source: Nat Commun. 2025 Dec 6;17:274. doi: 10.1038/s41467-025-66984-2 (PMC12783686; doi:10.1038/s41467-025-66984-2)
Supplement: Supplementary file 2 — Description of Additional Supplementary File [file 41467_2025_66984_MOESM2_ESM.pdf]

Description of Additional Supplementary Files

Supplementary Movie 1: in vitro fusion 1- Representative movie of fusion event of droplets containing mEGFP-β-catWt (green), mCherry-α-cat (magenta) and E-cadcyto -mTagBFP2 (cyan). The indicated proteins were premixed at a concentration of 1 µM before the addition of 10% PEG-8000. Time in minutes:seconds, scale bar represents 1 µm

Supplementary Movie 2: in vitro fusion 2- Representative movie of fusion event of droplets containing mEGFP-β-catWt (green), mCherry-α-cat (magenta) and E-cadcyto -mTagBFP2 (cyan). The indicated proteins were premixed at a concentration of 1 µM before the addition of 10% PEG-8000. Time in minutes:seconds, scale bar represents 1 µm.

Supplementary Movie 3: HCT116 mEGFP-β-catenin z-stack high density - Representative confocal z-stack (0.2 µm interval, looped) of endogenously tagged mEGFP-β-catenin in HCT116 cells at a confluent density. Scale bar represents 10 µm.

Supplementary Movie 4: HCT116 mEGFP-β-catenin z-stack low density - Representative confocal z-stack (0.2 µm interval, looped) of endogenously tagged mEGFP-β-catenin in HCT116 cells at low density, showing clusters at the free membrane. Scale bar represents 10 µm.

Supplementary Movie 5: mESC mEGFP-β-catenin z-stack - Representative confocal z-stack (0.2 µm interval, looped) of endogenously tagged mEGFP-β-catenin in mouse embryonic stem cells (mESCs). Scale bar represents 10 µm.

Supplementary Movie 6: HEK293t mEGFP-β-catenin z-stack - Representative confocal z-stack (0.2 µm interval, looped) of endogenously tagged mEGFP-β-catenin in HEK293t cells. Scale bar represents 10 µm.

Supplementary Movie 7: HCT116 mEGFP-β-catenin fusion - Representative time-lapse imaging of endogenously tagged mEGFP-β-catenin in HCT116 cells showing the coalescence of two clusters. Time in hours:minutes:seconds; scale bar represents 1 µm.

Supplementary Movie 8: HCT116 mEGFP-β-catenin CytoD - Representative time-lapse imaging of endogenously tagged mEGFP-β-catenin in HCT116 cells upon the addition of 2 µg/ml Cytochalasin D. Time in hours:minutes:seconds since the addition of Cytochalasin D; scale bar represents 10 µm.

Supplementary Movie 9: HCT116 mEGFP-β-catenin FRAP cell-cell - Representative time-lapse imaging of Fluorescent Recovery After Photobleaching (FRAP) experiment. A single mEGFP-β-catenin cluster at a cell-cell contact of endogenously tagged HCT116 cells was photobleached (area indicated with yellow box, time point 0) and imaged over time. Time in hours:minutes:seconds; scale bar represents 1 µm.

Supplementary Movie 10: HCT116 mEGFP-β-catenin FRAP free membrane - Representative time-lapse imaging of Fluorescent Recovery After Photobleaching (FRAP) experiment. A single mEGFP-β-catenin cluster at the free membrane of endogenously tagged HCT116 cells was photobleached (area indicated with yellow box, time point 0) and imaged over time. Time in hours:minutes:seconds; scale bar represents 1 µm.

Supplementary Movie 11: MDCK junction formation Wt - Representative time-lapse movie of normal de novo contact formation in β-catenin knock-out (KO) MDCK cells with mSc-βcateninWt addback (Fire LUT). Time in hours:minutes:seconds since the initial contact formation, scalebar represents 10 µm

Supplementary Movie 12: MDCK junction formation cIDR - Representative time-lapse movie showing clustering defects in β-catenin knock-out (KO) MDCK cells with mSc-β-catenincIDR* addback (Fire LUT) during de novo contact formation. Time in hours:minutes:seconds since the initial contact formation, scalebar represents 10 µm.

Supplementary Movie 13: MDCK junction formation IDRs - Representative time-lapse movie demonstrating a less efficient de novo contact formation in β-catenin knock-out (KO) MDCK cells with mSc-β-cateninIDRs* addback (Fire LUT). Both novel contacts show a reduced level of β-catenin clustering and a delay in cortical enrichment, whereas the right contact fails to enrich β-catenin entirely. Time in hours:minutes:seconds since the initial contact formation, scalebar represents 10 µm.

Supplementary Movie 14: MDCK junction formation nIDR - Representative time-lapse movie showing clustering defects and a failure to form a linear junction in β-catenin knock-out (KO) MDCK cells with mSc-β-cateninnIDR* addback (Fire LUT) during de novo contact formation. Time in hours:minutes:seconds since the initial contact formation, scalebar represents 10 µm.

Supplementary Movie 15: MDCK junction formation Wt - Representative time-lapse movie of normal de novo contact formation in β-catenin knock-out (KO) MDCK cells with mSc-βcateninWt addback (Fire LUT). Time in hours:minutes:seconds since the initial contact formation, scalebar represents 10 µm.

Supplementary Movie 16: MDCK junction formation cIDR - Representative time-lapse movie showing a delay in enrichment in β-catenin knock-out (KO) MDCK cells with mSc-β-catenincIDR* addback (Fire LUT) during de novo contact formation. Time in hours:minutes:seconds since the initial contact formation, scalebar represents 10 µm.

Supplementary Movie 17: MDCK junction formation no linearization - Representative time-lapse movie showing a failure to establish a linear cell-cell contact and instead maintain their dynamic protrusions in β-catenin knock-out (KO) MDCK cells with mSc-β-catenincIDR* addback (Fire LUT). Time in hours:minutes:seconds since the initial contact formation, scalebar represents 10 µm.

Supplementary Movie 18: MDCK junction formation and breakage - Representative time-lapse movie showing an unsuccessful de novo contact formation and instead junction breakage in β-catenin knock-out (KO) MDCK cells with mSc-βcatenincIDR* addback (Fire LUT). Time in hours:minutes:seconds since the initial contact formation, scalebar represents 10 μm.
