# Supplementary material for: β-catenin condensation facilitates clustering of the cadherin/catenin complex and formation of nascent cell-cell junctions
Source: Nat Commun. 2025 Dec 6;17:274. doi: 10.1038/s41467-025-66984-2 (PMC12783686; doi:10.1038/s41467-025-66984-2)
Supplement: Supplementary file 21 — Reporting summary [file 41467_2025_66984_MOESM21_ESM.pdf]

nature portfolio

Corresponding author(s): Jurian Schuijers and Martijn Gloerich

Last updated by author(s): Oct 23, 2026

Reporting Summary

Nature Portfolio wishes to improve the reproducibility of the work that we publish. This form provides structure for consistency and transparency in reporting. For further information on Nature Portfolio policies, see our Editorial Policies and the Editorial Policy Checklist.

Statistics

For all statistical analyses, confirm that the following items are present in the figure legend, table legend, main text, or Methods section.

n/a	Confirmed	
☐	☒	The exact sample size (*n*) for each experimental group/condition, given as a discrete number and unit of measurement
☐	☒	A statement on whether measurements were taken from distinct samples or whether the same sample was measured repeatedly
☐	☒	The statistical test(s) used AND whether they are one- or two-sided<br>*Only common tests should be described solely by name; describe more complex techniques in the Methods section.*
☒	☐	A description of all covariates tested
☐	☒	A description of any assumptions or corrections, such as tests of normality and adjustment for multiple comparisons
☐	☒	A full description of the statistical parameters including central tendency (e.g. means) or other basic estimates (e.g. regression coefficient) AND variation (e.g. standard deviation) or associated estimates of uncertainty (e.g. confidence intervals)
☐	☒	For null hypothesis testing, the test statistic (e.g. *F*, *t*, *r*) with confidence intervals, effect sizes, degrees of freedom and *P* value noted<br>*Give P values as exact values whenever suitable.*
☒	☐	For Bayesian analysis, information on the choice of priors and Markov chain Monte Carlo settings
☒	☐	For hierarchical and complex designs, identification of the appropriate level for tests and full reporting of outcomes
☐	☒	Estimates of effect sizes (e.g. Cohen's *d*, Pearson's *r*), indicating how they were calculated

Our web collection on statistics for biologists contains articles on many of the points above.

Software and code

Policy information about availability of computer code

Data collection	All fluorescent imaging data was aquired using NIS-Elements software, Zen image acquisition software or LAS X software. Electron microscopy data was acquired using SerialEM software.
Data analysis	Droplet formation assays were analyzed with Python using a previously published pipeline (DOI: 10.1016/j.cell.2020.11.030), all other imaging data was analysed using ImageJ. All statistical analyses were performed using Prism 8 software (GraphPad) and R.

For manuscripts utilizing custom algorithms or software that are central to the research but not yet described in published literature, software must be made available to editors and reviewers. We strongly encourage code deposition in a community repository (e.g. GitHub). See the Nature Portfolio guidelines for submitting code & software for further information.

Data

Policy information about availability of data

All manuscripts must include a data availability statement. This statement should provide the following information, where applicable:

- Accession codes, unique identifiers, or web links for publicly available datasets
- A description of any restrictions on data availability
- For clinical datasets or third party data, please ensure that the statement adheres to our policy

Source data are provided with this paper. Original imaging data, plasmids and cell lines are available upon request.

Research involving human participants, their data, or biological material

Policy information about studies with human participants or human data. See also policy information about sex, gender (identity/presentation), and sexual orientation and race, ethnicity and racism.

Reporting on sex and gender	n/a
Reporting on race, ethnicity, or other socially relevant groupings	n/a
Population characteristics	n/a
Recruitment	n/a
Ethics oversight	n/a

Note that full information on the approval of the study protocol must also be provided in the manuscript.

Field-specific reporting

Please select the one below that is the best fit for your research. If you are not sure, read the appropriate sections before making your selection.

☒ Life sciences ☐ Behavioural & social sciences ☐ Ecological, evolutionary & environmental sciences

For a reference copy of the document with all sections, see nature.com/documents/nr-reporting-summary-flat.pdf

Life sciences study design

All studies must disclose on these points even when the disclosure is negative.

Sample size	No statistical methods were used to predetermine sample size. Samples sizes are indicated in the legend of each experiment and were chosen to include sufficient numbers to achieve statistical power.
Data exclusions	For the selection of cell-cell contacts for analyses, only cells showing a proper expression of the transfected constructs were included. For the comparison between wildtype and mutant proteins, we analyze cell-cell contacts showing comparable expression levels.
Replication	All data are representative of or analyzed from at least three representative biological replicates, with the exception of Figure 3CD (one), 5A (two) and 5EF (two). All replicates gave similar results.
Randomization	n/a
Blinding	Data analysis was either performed using automated image analysis; or blinded when possible when it was manually analyzed.

Reporting for specific materials, systems and methods

We require information from authors about some types of materials, experimental systems and methods used in many studies. Here, indicate whether each material, system or method listed is relevant to your study. If you are not sure if a list item applies to your research, read the appropriate section before selecting a response.

Materials & experimental systems

n/a	Involved in the study	
☐	☒	Antibodies
☐	☒	Eukaryotic cell lines
☒	☐	Palaeontology and archaeology
☒	☐	Animals and other organisms
☒	☐	Clinical data
☒	☐	Dual use research of concern
☒	☐	Plants

Methods

n/a	Involved in the study	
☒	☐	ChIP-seq
☒	☐	Flow cytometry
☒	☐	MRI-based neuroimaging

Antibodies

Antibodies used	The following commercial antibodies were used at the indicated concentrations for Western blot (WB) and immunofluorescence (IF): rabbit anti-αE-catenin (Sigma-Aldrich; C2081; 1:500 IF); rabbit anti-β-catenin (Sigma; C2206; 1:2500 WB); mouse anti-β-catenin (BD

	Biosciences; 9018884; 1:1000 IF); mouse anti-α-tubulin (DM1A; Calbiochem; CP06; 1:5000 WB); rat anti-RFP (5F8; Chromotek; 1:2500 WB); and mouse anti-γ-catenin (Plakoglobin; Zymed; 13-8500 1:250 IF). For electron microscopy, biotin-anti-GFP (Rockland; 600-106-215; 1:300) and rabbit anti-biotin (Rockland; 100-4198; 1:10 000) antibodies were used.
Validation	All antibodies were obtained from commercial suppliers. Specificity of anti-αE-catenin, anti-β-catenin and anti-γ-catenin antibodies was validated in knockout cells (doi: 10.1091/mbc.E16-12-0851, current manuscript, and by the supplier, respectively). Specificity of anti-RFP and anti-GFP antibodies was validated by comparison to cells lacking expression of RFP- and GFP-tagged constructs.

Eukaryotic cell lines

Policy information about cell lines and Sex and Gender in Research

Cell line source(s)	HEK293T and HCT116 cells were derived from ATCC (CRL-3216 and CCL-247, respectively). mESC V6.5 were a gift from Rudolf Jaenisch (Whitehead Institute), MDCK cells were gift from W.J. Nelson (Stanford University).
Authentication	Authentication was based on morphological features of the cell lines
Mycoplasma contamination	All cell lines were regularly tested negative for mycoplasm contamination
Commonly misidentified lines (See ICLAC register)	n.a.

Plants

Seed stocks	*Report on the source of all seed stocks or other plant material used. If applicable, state the seed stock centre and catalogue number. If plant specimens were collected from the field, describe the collection location, date and sampling procedures.*
Novel plant genotypes	*Describe the methods by which all novel plant genotypes were produced. This includes those generated by transgenic approaches, gene editing, chemical/radiation-based mutagenesis and hybridization. For transgenic lines, describe the transformation method, the number of independent lines analyzed and the generation upon which experiments were performed. For gene-edited lines, describe the editor used, the endogenous sequence targeted for editing, the targeting guide RNA sequence (if applicable) and how the editor was applied.*
Authentication	*Describe any authentication procedures for each seed stock used or novel genotype generated. Describe any experiments used to assess the effect of a mutation and, where applicable, how potential secondary effects (e.g. second site T-DNA insertions, mosiacism, off-target gene editing) were examined.*
